# TLE4 downregulation identified by WGCNA and machine learning algorithm promotes papillary thyroid carcinoma progression via activating JAK/STAT pathway

**DOI:** 10.7150/jca.95501

**Published:** 2024-07-09

**Authors:** Junyu Lin, Beichen Cai, Qian Lin, Xinjian Lin, Biao Wang, Xiangjin Chen

**Affiliations:** 1 Department of Thyroid and Breast Surgery, the First Affiliated Hospital, Fujian Medical University, 350005, Fuzhou, Fujian, China.; 2 Department of Thyroid and Breast Surgery, National Regional Medical Center, Binhai Campus of the First Affiliated Hospital, Fujian Medical University, 350212, Fuzhou, Fujian, China.; 3 Department of Plastic Surgery, the First Affiliated Hospital of Fujian Medical University, 350005, Fuzhou, Fujian, China.; 4 Department of Plastic Surgery, National Regional Medical Center, Binhai Campus of the First Affiliated Hospital, Fujian Medical University, 350212, Fuzhou, Fujian, China.; 5 Key Laboratory of Gastrointestinal Cancer, Fujian Medical University, Ministry of Education, 350108, Fuzhou, Fujian, China.

**Keywords:** Papillary Thyroid Carcinoma (PTC), TLE4, JAK/STAT signaling pathway, Weighted Gene Co-expression Network Analysis (WGCNA), Machine learning algorithms

## Abstract

**Background:** Papillary Thyroid Carcinoma (PTC), a common type of thyroid cancer, has a pathogenesis that is not fully understood. This study utilizes a range of public databases, sophisticated bioinformatics tools, and empirical approaches to explore the key genetic components and pathways implicated in PTC, particularly concentrating on the Transducin-Like Enhancer of Split 4 (TLE4) gene.

**Methods:** Public databases such as TCGA and GEO were utilized to conduct differential gene expression analysis in PTC. Hub genes were identified using Weighted Gene Co-expression Network Analysis (WGCNA), and machine learning techniques, including Random Forest, LASSO regression, and SVM-RFE, were employed for biomarker identification. The clinical impact of the TLE4 gene was assessed in terms of diagnostic accuracy, prognostic value, and its functional enrichment analysis in PTC. Additionally, the study focused on understanding the role of TLE4 in the dynamics of immune cell infiltration, gene function enhancement, and behaviors of PTC cells like growth, migration, and invasion. To complement these analyses, *in vivo* studies were performed using a xenograft mouse model.

**Results:** 244 genes with significant differential expression across various databases were identified. WGCNA indicated a strong link between specific gene modules and PTC. Machine learning analysis brought the TLE4 gene into focus as a key biomarker. Bioinformatics studies verified that TLE4 expression is lower in PTC, linking it to immune cell infiltration and the JAK-STAT signaling pathways. Experimental data revealed that decreased TLE4 expression in PTC cell lines leads to enhanced cell growth, migration, invasion, and activates the JAK/STAT pathway. In contrast, TLE4 overexpression in these cells inhibited tumor growth and metastasis.

**Conclusions:** This study sheds light on TLE4's crucial role in PTC pathogenesis, positioning it as a potential biomarker and target for therapy. The integration of multi-omics data and advanced analytical methods provides a robust framework for understanding PTC at a molecular level, potentially guiding personalized treatment strategies.

## 1. Introduction

Papillary thyroid carcinoma (PTC), distinguished as the predominant form of thyroid malignancy, is characterized by its overall favorable prognosis 1, 2. Nevertheless, the comprehensive molecular underpinnings of this disease remain elusive, underscoring a critical gap in our understanding. Recent advances in the fields of genomics and transcriptomics have revolutionized our approach towards elucidating the genetic landscape of PTC 3-5. The specificity of these advances lies in the development and application of high-resolution sequencing technologies, such as next-generation sequencing (NGS) and single-cell RNA sequencing (scRNA-seq), which have provided unprecedented insights into the genomic and transcriptomic complexities of PTC. These methodologies have enabled the detailed characterization of gene expression patterns, genomic alterations, and epigenetic modifications at a resolution previously unattainable. This focus on numerous candidates has been informed by a nuanced understanding of its genetic context, derived from comprehensive data integration and analysis of large-scale genomic datasets, such as those available in TCGA and GEO. Such integrative approaches underscore the value of advanced bioinformatics tools in discerning the molecular signatures that distinguish PTC from other malignancies.

These technological strides have facilitated a more nuanced comprehension of the disease's genetic intricacies, spotlighting the pivotal role of differential gene expression and molecular pathways in the pathogenesis of PTC 6-8. Such insights are imperative for the delineation of novel diagnostic markers and therapeutic avenues, propelling the frontier of personalized medicine in oncology.

The advent of high-throughput genomic technologies has markedly shifted the paradigm in cancer research, heralding a new era of precision oncology, particularly in the context of PTC 9-11. The methodical scrutiny of public genomic repositories, including The Cancer Genome Atlas (TCGA) and the Gene Expression Omnibus (GEO), has been instrumental in the discovery of differentially expressed genes and the elucidation of complex regulatory networks underpinning PTC 12-14. These endeavors facilitate the identification of potential biomarkers and therapeutic targets, with the integration of data across multiple databases bolstering the robustness and reliability of these findings.

In this vein, our investigation employs a suite of sophisticated bioinformatics tools, encompassing Weighted Gene Co-expression Network Analysis (WGCNA), and an array of machine learning algorithms, such as Random Forest, Least Absolute Shrinkage and Selection Operator (LASSO) regression, and Support Vector Machine-Recursive Feature Elimination (SVM-RFE) 15-21. These methodologies are leveraged to pinpoint gene clusters and refine the selection of putative biomarkers within the PTC genomic landscape. Through this analytical rigor, we dissect the genetic complexity of PTC, unearthing pivotal genes that may play a crucial role in the disease's pathogenesis.

A focal point of our study is the Transducin-Like Enhancer of Split 4 (TLE4) gene, which emerges as a significant candidate through our analyses 22-25. Studies have demonstrated that TLE4 functions in a context-dependent manner, acting as a tumor suppressor or oncogene in different cancer types. For instance, TLE4's suppression has been linked to adverse outcomes in acute myeloid leukemia (AML), where it is thought to play a crucial role in hematopoietic differentiation and apoptosis. Conversely, in colorectal cancer, TLE4 has been implicated in promoting tumor progression through Wnt/β-catenin signaling. This multifaceted role of TLE4 across the cancer spectrum not only emphasizes the complexity of its function but also suggests a potential mechanism of action that could be leveraged in PTC. We delve into the diagnostic and prognostic implications of TLE4, alongside its influence on immune cell infiltration within the tumor microenvironment. Our objective is to underscore the prominence of TLE4 as a biomarker in PTC, elucidating its potential to inform clinical decision-making and therapeutic strategies.

To substantiate our bioinformatics findings, our study integrates a comprehensive array of *in vitro* and *in vivo* experiments, aimed at validating the functional relevance of TLE4 and other significant genes in the context of PTC. Techniques such as quantitative Polymerase Chain Reaction (qPCR), Western Blotting, and various cell assays are employed to affirm the downregulation of TLE4 in PTC and its conceivable role as a tumor suppressor. We explore the mechanism through which TLE4 modulates cellular proliferation and the Janus Kinase/Signal Transducer and Activator of Transcription (JAK/STAT) pathway, shedding light on novel molecular mechanisms implicated in PTC pathogenesis. This integrative approach, melding bioinformatics insights with empirical validation, significantly enhances our comprehension of TLE4's role in PTC, setting the stage for future research into targeted therapies and innovative management strategies for this disease.

This comprehensive and methodologically rigorous exploration not only enriches our understanding of PTC's molecular basis but also propels the development of innovative diagnostic and therapeutic interventions. By marrying advanced bioinformatics analyses with empirical research, we pave the way for advancements in the management of PTC, aspiring to improve patient outcomes through precision medicine and targeted therapy.

## 2. Materials and Methods

### 2.1 Database exploration and data extraction

In the initial phase of our study, we systematically explored various publicly available databases to extract data pertinent to PTC. This involved a detailed review of the Thyroid Carcinoma (THCA) dataset from The Cancer Genome Atlas (TCGA) and multiple datasets from the Gene Expression Omnibus (GEO), specifically GSE3467, GSE3678, GSE29265, GSE33630, and GSE60542. The inclusion of datasets was predicated upon the availability of high-quality, peer-reviewed, and publicly accessible gene expression profiles pertinent to papillary thyroid carcinoma (PTC) and matched normal thyroid samples. The selection criteria for these databases were stringent, focusing on datasets that provided a complete spectrum of data from both PTC and normal thyroid samples. This selection process was critical for ensuring a comprehensive and unbiased differential gene expression analysis. Each database was meticulously scrutinized, and the relevant data were extracted using standardized bioinformatics tools. The data extraction from the TCGA and GEO databases was facilitated by the use of the TCGAbiolinks (version 2.14.1) and GEOquery (version 2.56.0) packages in R, respectively. The selection of these specific tools was predicated on their widespread acceptance within the scientific community for ensuring robust and reliable data retrieval.

### 2.2 Differential gene expression analysis

For the analysis of differential gene expression (DEGs) in PTC, we employed robust bioinformatics tools. Initially, the extracted raw data from the databases underwent preprocessing, including normalization and quality control checks, to ensure data integrity. We then applied the limma package from Bioconductor, an R-based tool, for the identification of DEGs 26. The version of the limma package employed in our analyses was 3.42.2. Our choice of limma for differential expression analysis was informed by its well-documented efficacy in dealing with complex experimental designs and its ability to efficiently manage the statistical challenges associated with high-dimensional data. Limma's robust modeling framework, which incorporates empirical Bayes methods, ensures the reliability of our differential expression results. Alternative methods, such as DESeq2 and edgeR, were also considered; however, limma's unique empirical Bayes moderation of standard errors provides a more stable and powerful method for identifying DEGs in studies with the comparative dimension and diversity of ours.

The criteria for differential expression included an absolute log2 fold change (log2FC) greater than 1 and an adjusted P-value (Padj) less than 0.05. This stringent threshold was set to select genes with statistically significant and biologically relevant alterations in expression levels. Volcano plots were generated to visually represent the DEGs, contrasting statistical significance against fold-change. This visualization facilitated a clear and concise depiction of the genes markedly upregulated or downregulated in PTC compared to normal samples. Additionally, the results were tabulated to provide a quantitative summary of the DEGs identified in each dataset, allowing for a comprehensive comparative analysis across the different databases.

### 2.3 Intersecting gene analysis using upset plots

To further elucidate the complexity of differential gene expression across multiple datasets, we employed the UpSet plot analysis, a novel approach for intersecting gene analysis 27. This method, implemented using the R package “UpSetR”, allowed us to dissect and visualize the intersections of differentially expressed genes (DEGs) across the various PTC databases. In this analysis, upregulation and downregulation of the same gene were treated as distinct entities, ensuring accurate identification of consistent trends in expression changes across all databases. The UpSet plot provided a comprehensive view of the shared and unique DEGs among the datasets, enabling us to identify a core set of genes that demonstrated significant differential expression across all six PTC databases.

### 2.4 Weighted gene co-expression network analysis (WGCNA)

The next step in our research involved the application of Weighted Gene Co-expression Network Analysis (WGCNA) to identify hub genes associated with PTC. We used the WGCNA package in R to construct a scale-free co-expression network 28. WGCNA is not merely focused on individual gene associations but emphasizes the collective behavior of gene clusters, offering a more holistic view of gene interactions and their potential impact on PTC. The decision to utilize WGCNA was informed by its proven efficacy in uncovering biologically meaningful patterns, particularly in the context of complex diseases like cancer. Its capacity to construct a scale-free network aligns well with biological network properties, making it superior to alternative methods like hierarchical clustering and principal component analysis for our study objectives. Initially, we performed sample clustering to ensure the exclusion of outliers, thus maintaining the integrity of the network. The rationale for excluding these outliers was twofold: to prevent the skewing of network construction and to ensure that subsequent analyses were reflective of biologically relevant patterns inherent to the majority of the sample population.

The optimal soft-thresholding power was determined by ensuring the scale-free fit index approached 0.9, a crucial step for constructing a biologically meaningful network. The chosen power was the lowest value that allowed the scale-free topology fit index to reach at least 0.9, ensuring a genuine network topology while minimizing the potential for noise-induced artifacts. Modules of co-expressed genes were identified using hierarchical clustering, followed by the identification of modules most relevant to PTC traits by integrating modules with eigengene connectivity (feature factor) exceeding 0.75. The module membership (MM) and gene significance (GS) metrics were then calculated for each gene to quantitatively represent their correlation with the PTC traits. This dual-metric approach allowed for a nuanced correlation of modules with clinical traits, enhancing the interpretability of the WGCNA findings.

### 2.5 GO, DO, and KEGG functional enrichment analysis

The biological relevance of selected genes was investigated through functional enrichment analysis using the "clusterProfiler" package in R 29, 30. This analysis comprised Disease Ontology (DO) to pinpoint potential disease links, Gene Ontology (GO) to examine biological processes (BP), molecular functions (MF), and cellular components (CC), and Kyoto Encyclopedia of Genes and Genomes (KEGG) to outline significant signaling pathways. We took care to eliminate redundant GO terms to enhance interpretability. The DO analysis offered a view into possible disease associations, GO analysis facilitated understanding of cellular function roles, and KEGG pathways provided insights into biological process involvement.

To address the potential issue of redundancy among GO terms, which can obscure the interpretation of enrichment results, we employed the 'simplify' function within the 'clusterProfiler' package. This function uses a semantic similarity measure to group highly similar GO terms, allowing for the selection of a representative term from each group. We applied a significance threshold of p < 0.05 for all analyses, adjusting for multiple testing with the Bonferroni method to minimize false positives. Additionally, functional network plots were created to illustrate the connections among different functional categories, enriching our interpretation of the biological significance of the gene modules identified.

### 2.6 Machine learning algorithms for biomarker discovery

In our comprehensive approach to identify potential biomarkers for PTC, we employed three distinct machine learning algorithms: Random Forest, LASSO (Least Absolute Shrinkage and Selection Operator) regression, and Support Vector Machine-Recursive Feature Elimination (SVM-RFE). Each algorithm was applied to a curated set of 216 key genes previously identified through differential gene expression and WGCNA analyses. We utilized k-fold cross-validation, where the dataset was randomly divided into k equal-sized subsets. For each validation run, one subset was retained as the test set while the remaining subsets were used for training. This process was repeated k times, with each subset serving as the test set exactly once. The choice of k was determined based on a balance between computational efficiency and model validation rigor, ultimately settling on a 10-fold cross-validation approach.

For the Random Forest algorithm, we used the “randomForest” package in R to analyze gene importance, focusing on genes with a significance level exceeding a predefined threshold. In the LASSO regression, implemented using the “glmnet” package in R, we optimized the lambda (λ) value to enhance the model's precision in differentiating between thyroid papillary carcinoma tissues and normal thyroid tissues. Lastly, the SVM-RFE analysis, conducted using the “e1071” package in R, was employed to incrementally eliminate features, aiming to identify a subset of genes that achieved the highest classification accuracy.

The rationale behind the selection of these specific machine learning algorithms stems from their demonstrated efficacy in feature selection and classification tasks within the genomic data analysis domain. Random Forest is renowned for its robustness in handling high-dimensional data and its ability to provide insights into feature importance, making it invaluable for identifying genes critical to PTC pathogenesis. LASSO regression, with its regularization capabilities, excels in reducing the complexity of models by penalizing the absolute size of coefficients, thereby mitigating overfitting and enhancing model interpretability. This characteristic is particularly beneficial in distinguishing the most predictive genes from a vast dataset. SVM-RFE stands out for its iterative feature elimination process, which systematically refines the set of genes to those with the most significant classification power. This step-wise reduction aligns with our objective to pinpoint a compact, yet potent, subset of biomarkers.

### 2.7 Bioinformatics analysis of TLE4 gene in TCGA-THCA dataset

To elucidate the expression of TLE4 gene in thyroid carcinoma, we conducted an extensive bioinformatics analysis using the TCGA-THCA (Thyroid Carcinoma) dataset. This analysis encompassed three key aspects: diagnostic efficacy, prognostic value, and GSEA functional analysis. Diagnostic efficacy was assessed through Receiver Operating Characteristic (ROC) curve analysis, employing the “pROC” package in R, focusing on the sensitivity and specificity of TLE4 expression in distinguishing thyroid carcinoma from non-cancerous tissue samples. The prognostic value (Progress Free Interval, PFI) of TLE4 expression was investigated via Kaplan-Meier survival curves and Cox proportional hazards regression models, with an emphasis on hazard ratios to understand the relative risk associated with varying levels of TLE4 expression.

The GSEA (Gene Set Enrichment Analysis) functional analysis was performed to explore the biological pathways and processes potentially influenced by TLE4 expression levels in thyroid carcinoma. Utilizing the GSEA software, we analyzed the TCGA-THCA dataset to identify enriched gene sets in samples with high versus low TLE4 expression. This approach aimed to uncover the molecular mechanisms through which TLE4 may contribute to thyroid carcinoma pathogenesis and progression. Specifically, we focused on identifying key signaling pathways, cellular processes, and molecular functions that were significantly associated with TLE4 expression, thereby providing insights into its role in the biological behavior of thyroid carcinoma. The enrichment scores, normalized enrichment scores (NES), and false discovery rate (FDR) q-values were calculated to ensure the robustness and significance of our findings. The GSEA software version 4.0.3 was used for functional analysis, selected for its updated database and algorithmic efficiency in handling large genomic datasets like TCGA.

### 2.8 Immune cell infiltration correlation analysis

In our study, we conducted a detailed analysis of the correlation between the expression of TLE4 and immune cell infiltration in PTC using the TIMER2.0 database 31. This database offers a comprehensive resource for systematic analysis of immune infiltrates across diverse cancer types. We analyzed the correlation of TLE4 expression with various immune cell types, including Eosinophils, T helper cells, Th1 cells, Central Memory T cells (Tcm), and immature Dendritic Cells (iDC), among others, using sophisticated statistical methods. Additionally, we segregated 512 PTC samples from the TCGA-THCA dataset into high and low TLE4 expression cohorts, employing the R package “xCell” to compare immune cell infiltration between these groups.

### 2.9 Single-gene differential expression analysis and functional enrichment

For a more in-depth understanding of TLE4's role in PTC, we performed a single-gene differential expression analysis between patient groups characterized by high and low TLE4 expression in TCGA-THCA. Utilizing the R packages “DESeq2”, we set the threshold for significant differential genes at an absolute log2 fold change (log2FC) greater than 1 and an adjusted P-value (P.adj) less than 0.05. This stringent selection criterion was vital for ensuring the precision of our findings. Following the identification of differentially expressed genes, we embarked on a comprehensive Gene Ontology (GO) functional enrichment analysis using the 'clusterProfiler' package in R. This analysis offered a nuanced understanding of the functional implications of these genes, highlighting significant enrichment in functionalities related to the JAK-STAT signaling pathway and other critical pathways, such as Cytokine-cytokine receptor interaction and Neuroactive ligand-receptor interaction.

### 2.10 Lentiviral Vector Construction and Cell Transfection Protocols

To achieve the overexpression of target genes, constructs were meticulously engineered by integrating fragments of the full-length coding sequence of TLE4 (Homo sapiens, Accession NM_007005.6, with a coding sequence of 2322 base pairs and a GC content of 63.10%) into the pHBLV-CMV-MCS-3flag-EF1-ZsGreen-T2A-Puro vectors, sourced from Hanbio Biotechnology, China. These constructs were then introduced into 293T cells, utilizing the Lipofiter™ Transfection Kit (Hanbio Biotechnology, China) to facilitate the transfection process.

Subsequent to the transfection, lentiviruses engineered for overexpression were employed to infect PTC cell lines, ensuring the delivery and expression of the TLE4 gene. A critical phase of selection commenced three days post-transfection, involving the addition of 1μg/mL puromycin to the culture medium. This selective agent was instrumental in isolating cells that had successfully integrated the transfection constructs, thereby enabling the enrichment of a population of cells expressing the TLE4 gene. To ascertain the efficiency of overexpression maneuvers, rigorous quantitative analysis was conducted using RT-qPCR and western blotting assays.

### 2.11 Quantitative polymerase chain reaction (qPCR) and Western blot analysis of TLE4 expression

To empirically validate the functional implications of TLE4 in PTC, we conducted qPCR and Western blot analysis. These assays were designed to quantitatively assess mRNA and protein expression levels of TLE4 in PTC cell lines (TPC-1, IHH-4, and B-CPAP) compared to a normative control cell line (Nthy-ori 3-1). All cell lines were purchased from ProCell Co., LTD, Wuhan, China.

For qPCR, total RNA was extracted using TRIzol reagent and reverse transcribed to cDNA using a high-capacity cDNA reverse transcription kit. qPCR was performed using SYBR Green PCR Master Mix on a Real-Time PCR System. Relative expression levels were calculated using the 2-ΔΔCt method, with ACTB(β-actin) serving as an internal control. Primer sequences employed were: TLE4: Forward, 5'-CGACCTGAGCAAGATGTACCC-3'; Reverse, 5'-CGATCACAGGATTCGGAAATTGT-3'; ACTB: Forward, 5'-CTCCATCCTGGCCTCGCTGT-3'; Reverse, 5'-GCTGCTACCTTCACCGTTCC-3'.

For Western blot analysis, total protein was extracted, quantified, and separated by SDS-PAGE, followed by transfer to PVDF membranes. Membranes were incubated with primary antibodies against TLE4 (ab64833, Abcam) and β-actin (ab8227, Abcam) as a loading control, followed by appropriate secondary antibodies. Bands were visualized using enhanced chemiluminescence and quantified with image analysis software.

### 2.12 Assessment of TLE4 gene overexpression on PTC cell growth

To investigate the impact of TLE4 gene overexpression on the growth of PTC cells, we employed cell viability and colony formation assays. TPC-1 and IHH-4 cell lines were chosen for this analysis due to their relevance in PTC research. We first achieved overexpression of TLE4 in these cell lines through transfection with a TLE4-overexpressing vector, while a control vector was used for the control groups. The efficiency of TLE4 overexpression was confirmed via qPCR and WB assays. Cell viability was assessed using the CCK-8 assay, where cells were seeded in 96-well plates, and CCK-8 reagent was added post-transfection to measure metabolic activity as an indirect indicator of cell proliferation. For the colony formation assay, cells were plated in 6-well plates and allowed to grow until visible colonies formed. The colonies were then stained with crystal violet and quantified.

### 2.13 Analysis of TLE4-mediated inhibition of migration and invasion in PTC Cells

To examine the role of TLE4 in the migration and invasion of PTC cells, we conducted *in vitro* scratch and Transwell invasion assays. The scratch assay, also known as a wound healing assay, involved creating a linear “scratch” in a monolayer of PTC cells and observing the closure of this gap over a specified time period. The rate of gap closure served as a measure of cell migration. For the invasion assay, we utilized Transwell chambers coated with Matrigel to simulate the extracellular matrix. PTC cells with overexpressed TLE4 and control cells were seeded into the upper chamber, and their ability to invade through the Matrigel to the lower chamber was quantified.

### 2.14 Evaluation of TLE4 impact on JAK/STAT pathway in PTC cells

To investigate the influence of TLE4 overexpression on the JAK/STAT signaling pathway in PTC cells, we conducted qPCR/Western blot analysis and functional assays. qPCR and Western blot analysis was employed to assess the levels of key proteins in the JAK/STAT pathway, particularly JAK2 and STAT3, in PTC cell lines with TLE4 overexpression. Primer sequences employed were: JAK2: Forward, 5'-ATCCACCCAACCATGTCTTCC-3'; Reverse, 5'-ATTCCATGCCGATAGGCTCTG-3'; STAT3: Forward, 5'-ACCAGCAGTATAGCCGCTTC-3'; Reverse, 5'-GCCACAATCCGGGCAATCT-3'.

Cells were lysed, and protein concentrations were determined using a BCA protein assay kit. Equal amounts of protein were loaded onto SDS-PAGE gels, transferred to PVDF membranes, and probed with specific antibodies against JAK2 (ab108596, Abcam), STAT3 (ab68153, Abcam), and β-actin as a loading control. Enhanced chemiluminescence detection was used to visualize the bands. Additionally, we introduced the JAK2/STAT3 inhibitor AG490 (HY-12000, MedChemExpress) into our experimental setup. PTC cells with and without TLE4 overexpression were treated with AG490, and subsequent changes in cell proliferation, migration, and invasion were evaluated using CCK-8, scratch, and Transwell invasion assays, respectively.

### 2.15 *In vivo* evaluation of tumor growth suppression by TLE4

To validate the tumor-suppressive role of TLE4 in an *in vivo* setting, we established a subcutaneous xenograft model using BALB/c nude mice. The TPC-1 PTC cell line, either overexpressing TLE4 or containing the control vector, was injected subcutaneously into the mice. Tumor growth was monitored by measuring tumor volume and mass at regular intervals. Upon reaching the endpoint of the experiment, the mice were sacrificed, and tumors were excised for further analysis. The excised tumors were subjected to Western blot analysis to assess the expression levels of TLE4 and key proteins in the JAK/STAT pathway. Additionally, immunohistochemical staining was performed on tumor sections to evaluate the expression of proliferation markers, including KI-67 and PCNA.

### 2.16 Statistical analysis

For the *in vitro* and *in vivo* experiments, data were expressed as mean ± standard deviation (SD). The significance of differences between groups was determined using the Student's t-test for two-group comparisons or one-way ANOVA for multiple comparisons, followed by a post-hoc Tukey's test when appropriate. For the differential expression analysis, we employed the Student's t-test to compare mean expression levels between PTC and normal thyroid tissues, due to its appropriateness for analyzing two independent sample means. In cases where multiple group comparisons were necessary, as in the analysis of gene expression across different PTC stages, one-way ANOVA was utilized to discern any statistically significant differences among group means, followed by Tukey's Honest Significant Difference test to conduct pairwise comparisons without inflating the Type I error rate.

The Kaplan-Meier method with the log-rank test was used for survival analysis, and Cox proportional hazards regression models were employed to determine hazard ratios. In the bioinformatics analyses, differential expression analysis, and correlation studies, p-values were adjusted for multiple testing using the Benjamini-Hochberg method to control the false discovery rate. A p-value (or adjusted p-value) of less than 0.05 was considered statistically significant. All statistical analyses were performed using R software and appropriate packages for specific tests, ensuring the reliability and scientific rigor of our findings.

## 3. Results

### 3.1 Identification and intersection analysis of differential genes across multiple PTC databases

**Figure 1:**
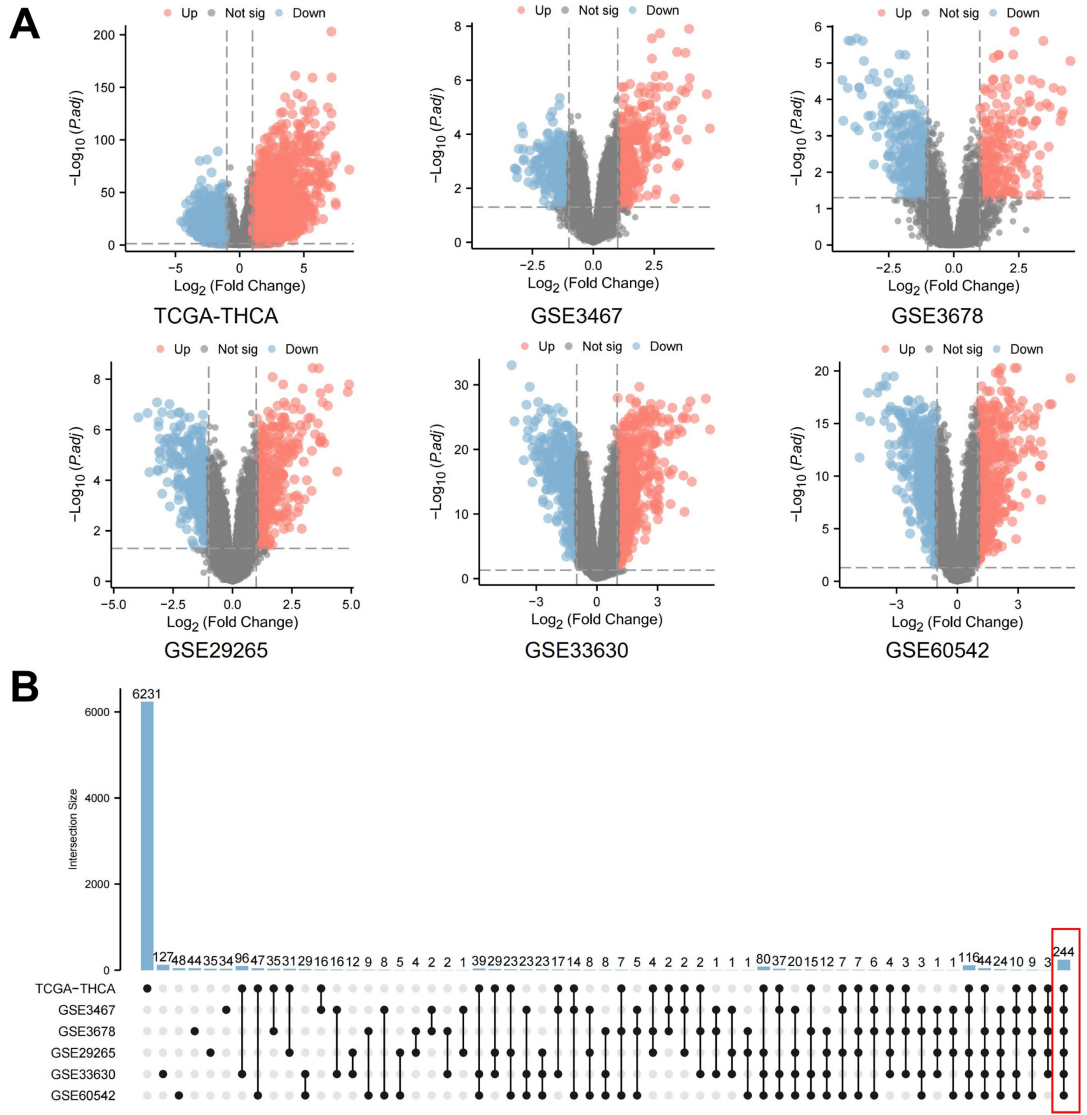
** Differential gene expression analysis in papillary thyroid carcinoma (PTC).** A. Volcano plots illustrating the statistical significance (adjusted P-value) versus fold-change in gene expression between PTC and normal thyroid samples across multiple databases. These plots highlight genes with an absolute log2 fold change (log2FC) over 1 and an adjusted P-value below 0.05. B. An UpSet plot depicting the intersection analysis of differentially expressed genes identified across the aforementioned databases. This analysis treats upregulation and downregulation of the same gene as distinct events to ensure consistent expression trends.

Initially, a thorough investigation of public databases for PTC was performed. This included the Thyroid Carcinoma (THCA) dataset from The Cancer Genome Atlas (TCGA) and various Gene Expression Omnibus (GEO) datasets (GSE3467, GSE3678, GSE29265, GSE33630, GSE60542). Data from both PTC and normal thyroid samples were meticulously gathered from each database for a comprehensive DEGs analysis. The volcano plots generated provide a visual comparison of statistical significance versus fold-change in gene expression (Figure 1A). Supplementary Table 1 concisely lists the number gene name of differentially expressed genes identified in each database, defined by an absolute log2 fold change (log2FC) over 1 and an adjusted P-value (Padj.) below 0.05. This strict selection criterion ensures the identification of genes with both statistically significant and biologically meaningful expression changes. Additionally, an UpSet plot analysis was conducted to examine the overlaps of differentially expressed genes across these databases (Figure 1B). This analysis uniquely considered upregulation and downregulation of the same gene as separate to confirm consistent expression trends across all databases. As a result, this thorough method identified a core set of 244 genes with significant differential expression in all six PTC databases, targeted for further detailed study.

### 3.2 Identification of hub genes using weighted gene co-expression network analysis (WGCNA)

**Figure 2:**
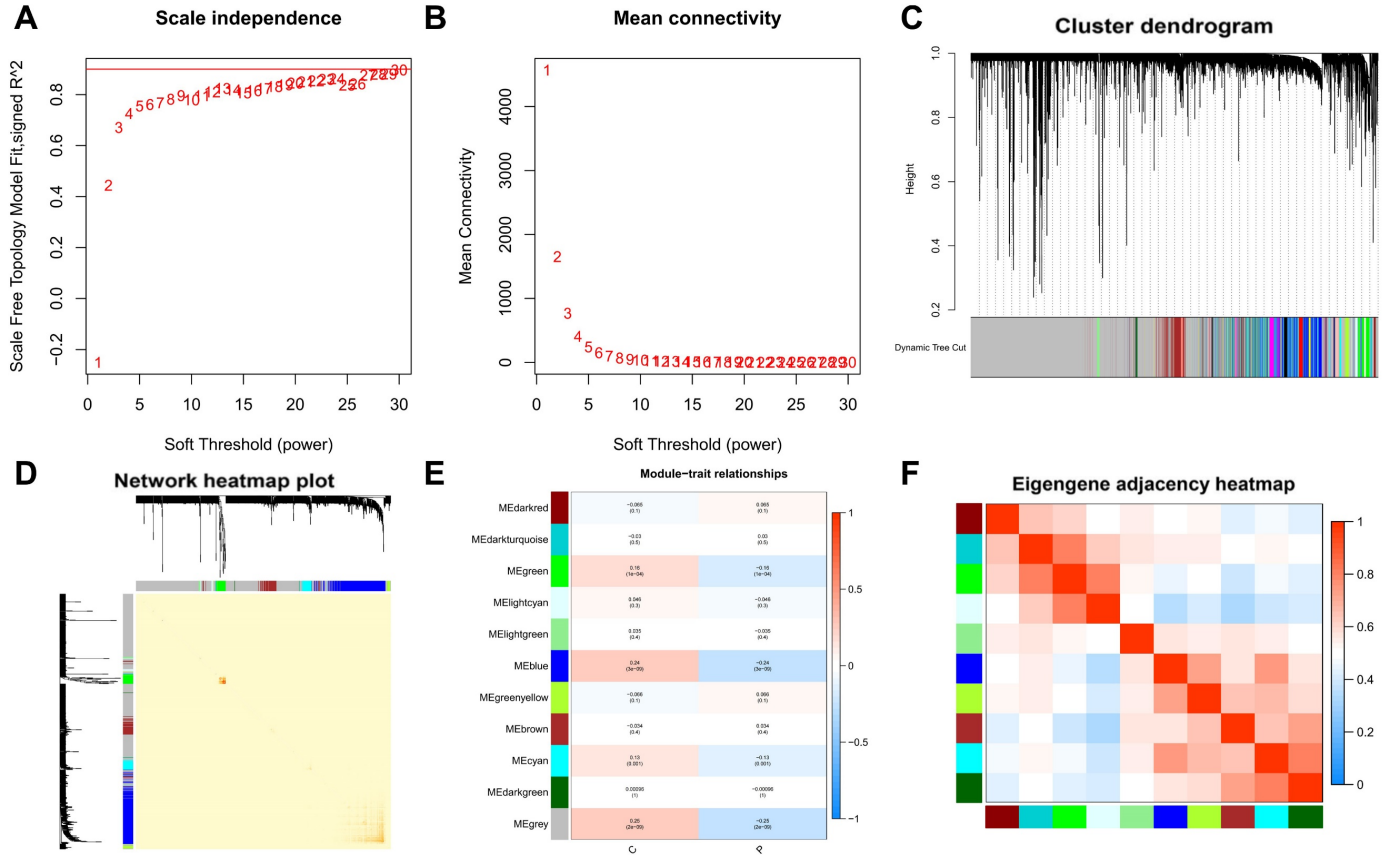
** Weighted gene co-expression network analysis (WGCNA) and identification of modules associated with PTC.** A-B. Determination of the optimal soft-thresholding power for scale-free network construction, demonstrated by achieving a scale-free fit index of 0.9. C-D. Visualization of module merging based on eigengene connectivity, with a threshold set at 0.75 to identify modules most relevant to PTC traits. E. Dendrogram and module-trait heatmap illustrating eleven distinct modules identified, with module membership (MM) and gene significance (GS) quantifying the correlation between gene expression and module eigengenes (MEs), as well as the link between samples and module genes, respectively. F. A focused view on the grey module's strong association with PTC, underscoring its potential in revealing key pathways or gene sets for PTC research.

In the clustering process, no outliers were identified, enhancing the reliability of our analysis. We determined the optimal soft-thresholding power for scale-free network construction by achieving a scale-free fit index of 0.9, essential for forming a scale-free topology in WGCNA (Figure 2A-B). This step ensures the network's alignment with biological system properties. We then merged modules with an eigengene connectivity exceeding 0.75 (Figure 2C-D), helping to pinpoint modules most relevant to our study traits. Eleven distinct modules were identified in the dendrogram and module-trait heatmap (Figure 2E), with module membership (MM) quantifying the correlation between gene expression and module eigengenes (MEs), and gene significance (GS) measuring the link between samples and module genes. Notably, the grey modules showed a strong association with PTC (Figure 2E-F), suggesting key pathways or gene sets for further PTC research. The statistical significance of these modules underscores their biological relevance.

**Figure 3:**
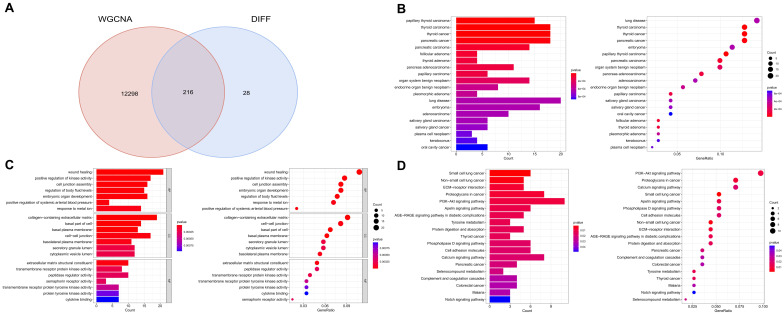
** Integration of WGCNA modules with differential expression analysis and subsequent pathway enrichment.** A. Intersection of module genes from WGCNA with previously identified differentially expressed genes (DEGs), focusing on genes central to the network and differentially expressed. B. Disease Ontology (DO) enrichment analysis demonstrating the strong association of these genes with thyroid malignancies, especially papillary thyroid carcinoma and thyroid carcinoma in general. C. Gene Ontology (GO) enrichment analysis highlighting the genes' predominant roles in wound healing and the positive regulation of kinase activity. D. KEGG pathway analysis identifying multiple signaling pathways associated with these genes, underscoring their relevance to the oncogenic processes in thyroid cancer.

By intersecting module genes from WGCNA with previously identified DEGs, we pinpointed 216 potential targets related to PTC (Figure 3A). This approach refines our target list, focusing on genes central to the network and differentially expressed in PTC. Subsequent enrichment analyses revealed significant biological processes and pathways associated with these genes. Disease Ontology (DO) enrichment analysis confirmed the strong association of these genes with thyroid malignancies, particularly papillary thyroid carcinoma and thyroid carcinoma in general (Figure 3B). Gene Ontology (GO) enrichment highlighted their predominant role in wound healing and the positive regulation of kinase activity, suggesting a potential influence on cell signaling and repair mechanisms in thyroid cancer pathophysiology (Figure 3C). Additionally, KEGG pathway analysis pinpointed the multiple signaling pathways, among others, underscoring their relevance to the oncogenic processes in thyroid cancer (Figure 3D). These findings elucidate the molecular underpinnings of papillary thyroid carcinoma and underscore the importance of these pathways in its development and progression.

### 3.3 Application of machine learning algorithms for biomarker screening in PTC patients

**Figure 4:**
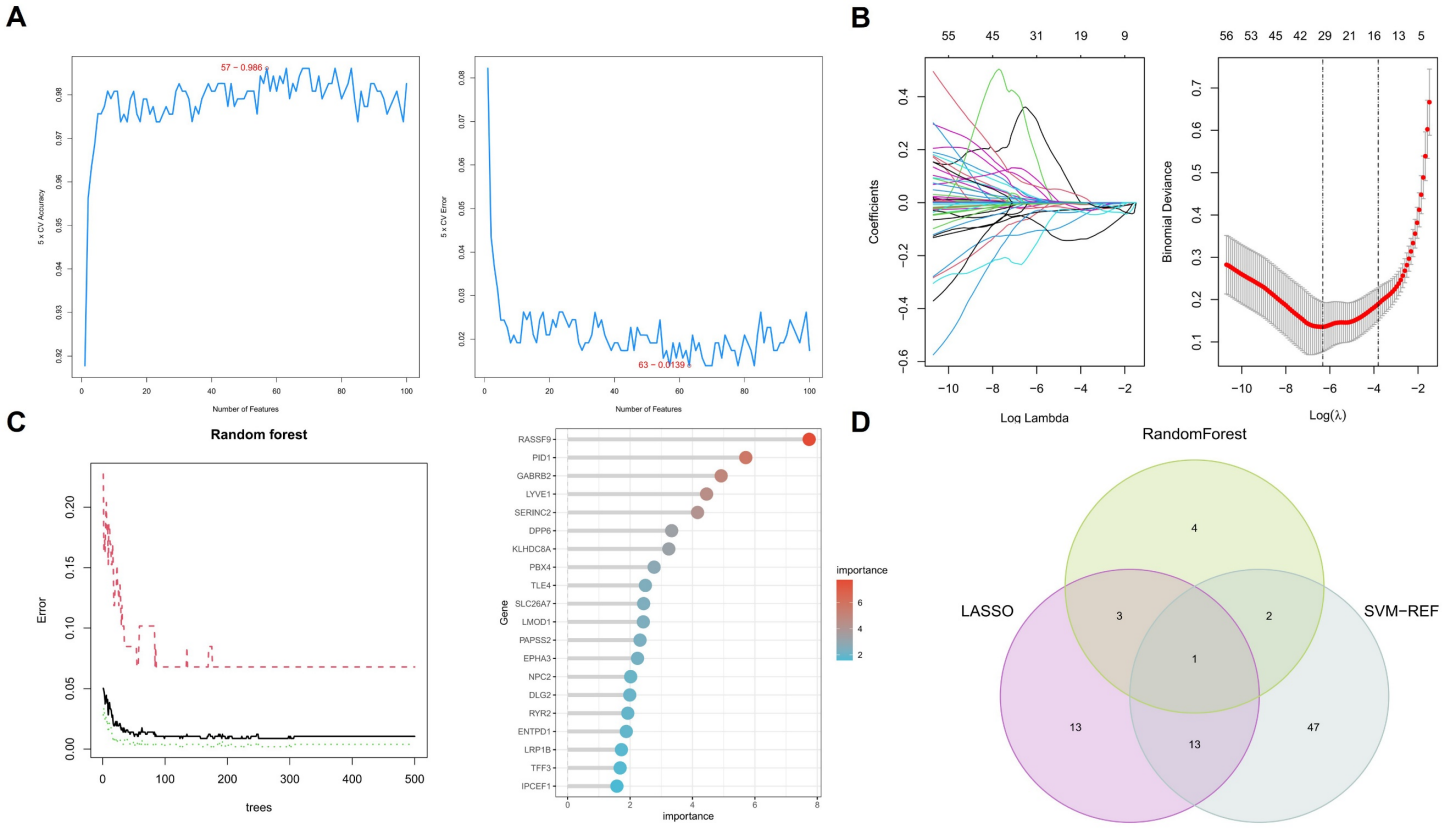
** Machine learning algorithms identify key biomarkers in papillary thyroid carcinoma (PTC).** A. Random Forest analysis identifying ten significant genes as prospective biomarkers for PTC. B. LASSO regression analysis distinguishing between thyroid papillary carcinoma and normal tissues, highlighting 30 potential genes with optimized lambda (λ) value settings. C. Support Vector Machine-Recursive Feature Elimination (SVM-RFE) analysis showcasing a model comprising 63 genes. D. Integration of findings from Random Forest, LASSO regression, and SVM-RFE analyses, emphasizing the TLE4 gene as a pivotal biomarker in PTC.

In this comprehensive study, we utilized three machine learning algorithms to examine 216 key genes for potential biomarkers in PTC. Employing Random Forest, we identified ten significant genes as prospective PTC biomarkers (Figure 4A), based on a stringent statistical threshold for reliability. We also used LASSO regression to distinguish between thyroid papillary carcinoma and normal tissues, pinpointing 30 potential genes with optimized lambda (λ) value settings (Figure 4B). Additionally, Support Vector Machine-Recursive Feature Elimination (SVM-RFE) analysis identified a model with 63 genes as most accurate (Figure 4C), highlighting PTC's complexity. The Random Forest model was tuned for optimal tree depth and number of trees, minimizing overfitting while maximizing predictive accuracy. The LASSO regression was applied with cross-validation to select the most informative genes, effectively reducing model complexity and enhancing interpretability. SVM-RFE, through its iterative feature elimination process, honed in on a subset of genes with maximal relevance to PTC pathology. This comprehensive application of machine learning models underscores the sophistication of our biomarker screening process, yielding insights of high potential utility for diagnostic and therapeutic development.

Integrating these algorithmic outputs, we singled out the TLE4 gene as a key biomarker linked to PTC (Figure 4D). This unified focus on TLE4, supported by multiple analytical methods, emphasizes its importance in PTC's pathology and potential as a therapeutic target. Overall, our findings offer a layered perspective on PTC's genetic landscape, identifying various biomarkers and culminating in the TLE4 gene, thereby enriching current knowledge and guiding future research in targeted therapies and diagnostics.

### 3.4 Comprehensive bioinformatics analysis of low-expressed TLE4 gene in TCGA-THCA

**Figure 5:**
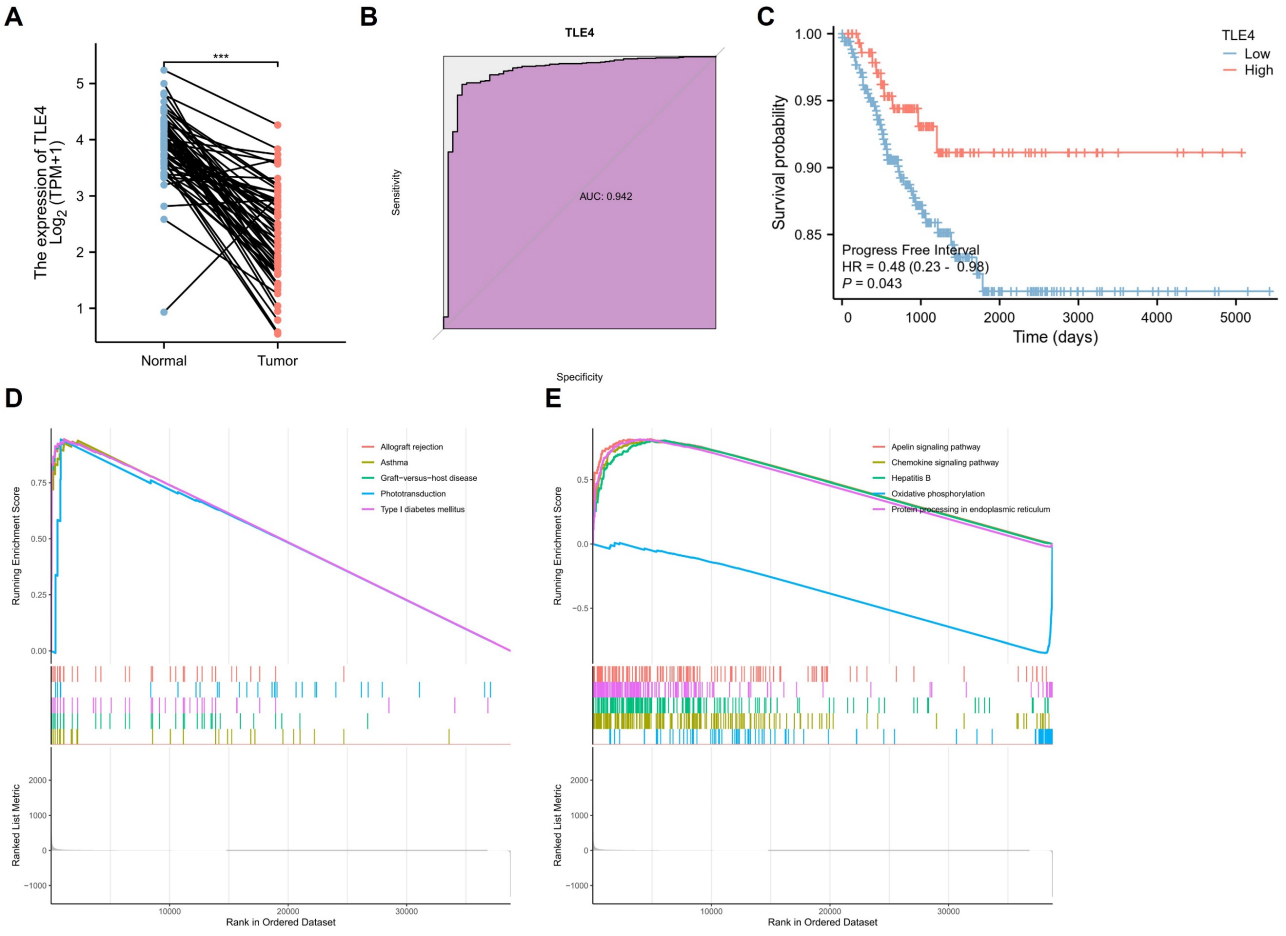
** Bioinformatics analysis of the TLE4 gene in thyroid carcinoma (TCGA-THCA) dataset.** A. Expression analysis of TLE4, demonstrating significantly lower expression levels in thyroid carcinoma compared to paired adjacent non-cancerous tissues. B. Receiver Operating Characteristic (ROC) curve analysis evaluating the diagnostic utility of TLE4 in differentiating thyroid carcinoma from non-cancerous tissues. C. Kaplan-Meier survival analysis and Cox regression models exploring the prognostic significance of TLE4, showing that patients with lower levels of TLE4 expression have a poorer prognosis. D. Gene Set Enrichment Analysis (GSEA) identifying pathways differentially enriched among upregulated genes in correlation with low TLE4 expression. E. GSEA of downregulated genes associated with TLE4 expression, highlighting enriched pathways that involve signaling mechanisms and cellular metabolism.

This section delves into an in-depth bioinformatics analysis of the TLE4 gene in thyroid carcinoma (TCGA-THCA), focusing on its diagnostic efficiency, prognostic potential, and GSEA functional analysis. Firstly, TLE4 expression was significantly different between thyroid carcinoma and paired adjacent tissues. Namely, TLE4 was significantly lower expressed in thyroid cancer (Figure 5A). Secondly, we assess TLE4's diagnostic utility by analyzing its ability to differentiate thyroid carcinoma from non-cancerous tissues using Receiver Operating Characteristic (ROC) curves (Figure 5B). The area under the curve (AUC) measures the diagnostic performance, with sensitivity (true positive rate) and specificity (true negative rate) evaluations helping to determine TLE4's effectiveness as a diagnostic biomarker (AUC = 0.942). The prognostic significance (Progress Free Interval, PFI) of TLE4 is explored through survival analysis, including Kaplan-Meier curves and Cox regression models (Figure 5C). That is, patients with low TLE4 levels have a worse prognosis. These analyses offer insights into the relationship between TLE4 expression and patient outcomes, with hazard ratios indicating the risk of adverse events linked to different TLE4 expression levels.

Lastly, in the TCGA-THCA dataset, GSEA identified distinct pathways differentially enriched in correlation with TLE4 expression. Among the upregulated genes, significant enrichment was observed for gene sets related to allograft rejection, asthma, graft-versus-host disease, phototransduction, and type I diabetes mellitus, indicating a potential involvement of TLE4 in immune response and metabolic processes (Figure 5D). Conversely, the analysis of downregulated genes revealed enrichment in pathways such as the apelin signaling pathway, chemokine signaling pathway, hepatitis B, oxidative phosphorylation, and protein processing in the endoplasmic reticulum (Figure 5E). This suggests a complex role for TLE4 in modulating signaling mechanisms and cellular metabolism. The enrichment plots display a pronounced deviation from the expected random distribution, underscoring the biological relevance of these pathways in the context of TLE4 expression in thyroid carcinoma. In conclusion, this thorough bioinformatics analysis enhances our understanding of TLE4's role in thyroid carcinoma, providing valuable insights into its potential as a biomarker for thyroid cancer management.

### 3.5 Correlation of TLE4 with immune cell infiltration in PTC

**Figure 6:**
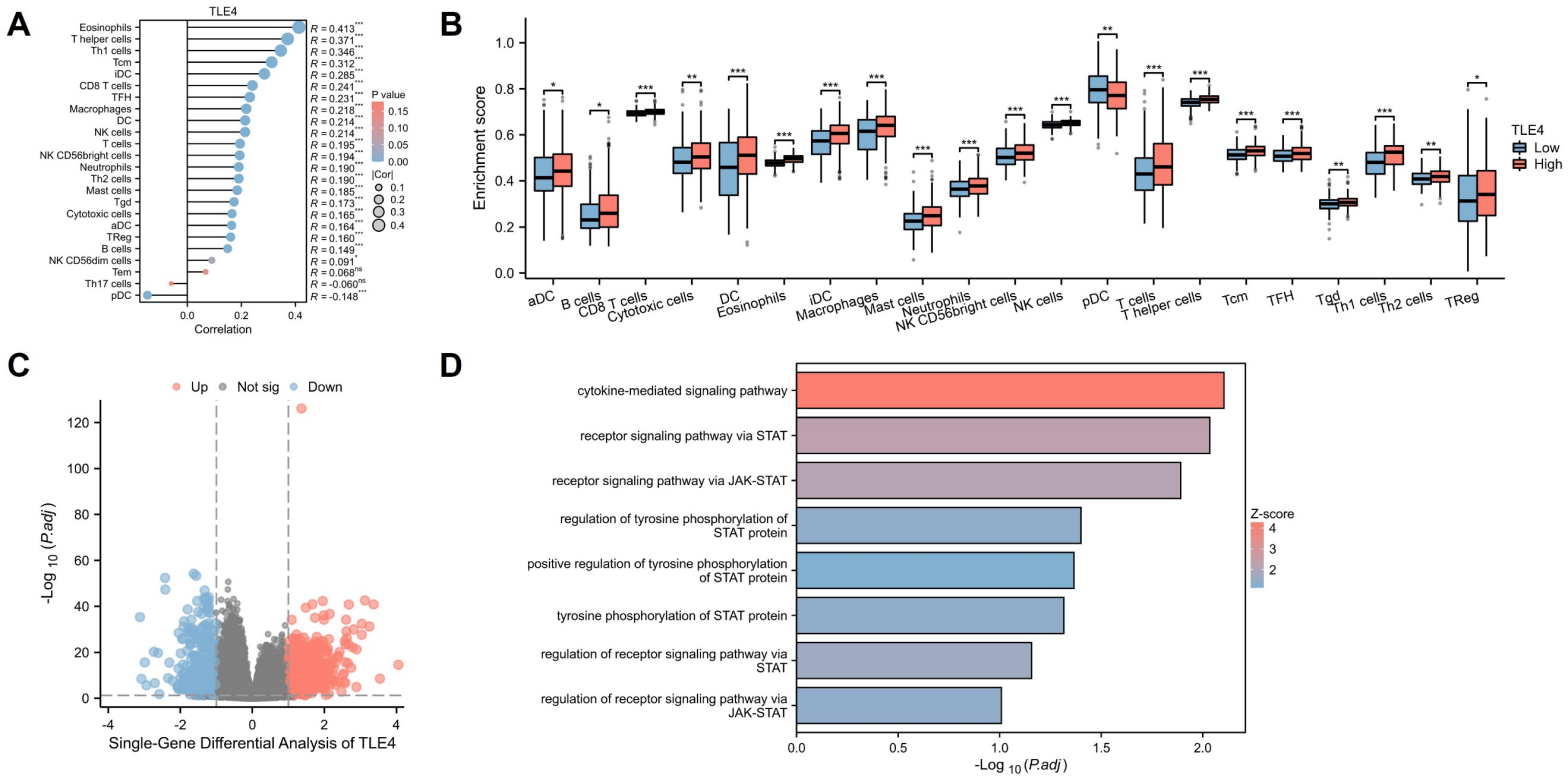
** Correlation of TLE4 expression with immune cell infiltration in PTC.** A. Analysis of the TIMER2.0 database illustrating a strong positive correlation between TLE4 expression levels and the infiltration of various immune cells, including eosinophils, T helper cells, and Th1 cells, while showing a negative correlation with plasmacytoid dendritic cells in the PTC tumor microenvironment. B. Stratification of 512 PTC samples from the TCGA-THCA dataset into high and low TLE4 expression groups, demonstrating significant differences in immune cell infiltration patterns, as analyzed using the R package xCell. C. Single-gene differential expression analysis between high and low TLE4 expression groups in PTC, employing strict selection criteria (|log2FC| > 1) to identify significantly altered genes. D. GO functional enrichment analysis revealing TLE4's significant association with the JAK-STAT signaling pathway, receptor signaling via STAT, and cytokine interactions.

PTC tumor microenvironment, significantly influenced by immune cell infiltration, is a critical genetic and prognostic marker for lymph node metastasis. We used the TIMER2.0 database to explore the relationship between TLE4 expression and immune cells in PTC. We found a strong positive correlation between TLE4 levels and various immune cells, including eosinophils, T helper cells, and Th1 cells and so on, and a negative correlation with plasmacytoid dendritic cells (Figure 6A). Dividing 512 PTC samples from the TCGA-THCA dataset into high and low TLE4 expression groups, we observed significant differences in immune cell infiltration using the R package xCell (Figure 6B). The intricate dynamics of TLE4 expression and immune cell infiltration underscore a complex interplay within the tumor microenvironment, which is pivotal for understanding PTC's progression and metastasis. The analysis of immune cell types, using algorithms like CIBERSORT, provides a granular view of the tumor immunology influenced by TLE4 expression levels, offering novel insights into how TLE4 may modulate immune evasion or activation in PTC.

We also conducted a single-gene differential analysis between these groups (Figure 6C), setting stringent criteria for significant gene identification (|log2FC| > 1). This was followed by an elaborate GO functional enrichment analysis (Figure 6D), revealing significant involvement in the JAK-STAT signaling pathway and other functions like receptor signaling via STAT and cytokine interactions, suggesting links to neuroendocrine activity and immune dysregulation in PTC. Overall, this analysis highlights TLE4's multifaceted role in PTC, pointing to possible targeted therapies and novel therapeutic targets by understanding the molecular mechanisms at play in PTC's progression.

### 3.6 TLE4 is downregulated in PTC cells

**Figure 7:**
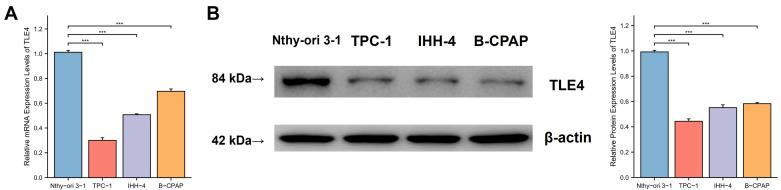
** TLE4 downregulation in PTC cell lines.** A. Quantitative PCR (qPCR) analysis showing significant reduction in TLE4 mRNA levels in PTC cell lines (TPC-1, IHH-4, B-CPAP) compared to the Nthy-ori 3-1 control cell line. B. Western blot analysis confirming a substantial decrease in TLE4 protein levels in the same PTC cell lines.

To validate the functional role of the TLE4 gene suggested by previous analyses, we conducted empirical research using qPCR) and Western blot analysis to examine TLE4 in PTC. As shown in Figures 7A and 7B, there was a significant reduction in TLE4 mRNA and protein levels in PTC cell lines (TPC-1, IHH-4, B-CPAP) compared to the Nthy-ori 3-1 control cell line, with statistical analyses confirming this substantial decrease. The pronounced difference in TLE4 expression between PTC and control cells points to TLE4's potential significance in thyroid cancer, encouraging further study into its regulatory functions and impact on thyroid carcinogenesis.

### 3.7 TLE4 expression inhibits PTC cell proliferation

**Figure 8:**
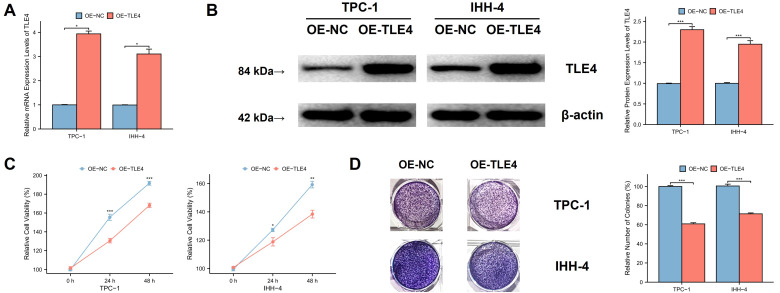
** TLE4 expression and its impact on PTC cell proliferation.** A. qPCR results indicating a notable increase in TLE4 mRNA levels following overexpression in TPC-1 and IHH-4 cell lines. B. Western blot analysis showing increased TLE4 protein levels upon overexpression in the same cell lines. C. Cell viability assays demonstrating a significant reduction in cell growth in TLE4-overexpressed PTC cell lines. D. Colony formation assays revealing fewer colonies in TLE4 overexpressed cells.

TPC-1 and IHH-4 cell lines were used to assess the effect of TLE4 gene expression on PTC cell growth. Significantly, TLE4 overexpression led to a notable increase in TLE4 mRNA and protein levels, confirming the effectiveness of our overexpression methods (Figure 8A and 8B). We observed a considerable reduction in cell viability in the TLE4-overexpressed cells (Figure 8C), indicating a link between higher TLE4 levels and decreased cell proliferation. Furthermore, colony formation assays showed fewer colonies in the TLE4 overexpressed cells (Figure 8D), supporting the idea that increased TLE4 expression impedes PTC cell proliferation. These results collectively suggest that TLE4's elevated expression significantly inhibits PTC cell growth.

### 3.8 TLE4 inhibits migration and invasion of PTC cells

**Figure 9:**
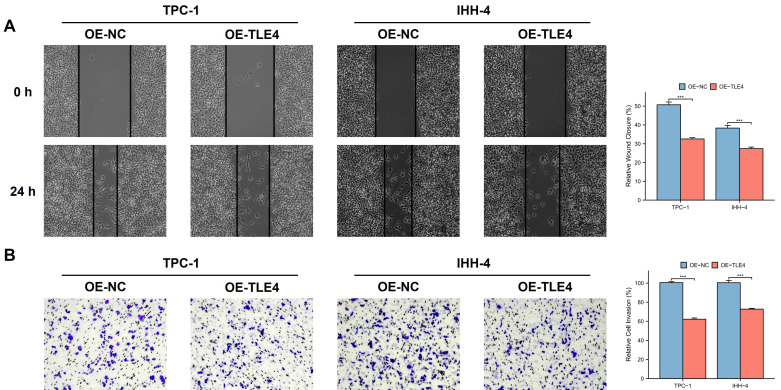
** TLE4 inhibits migration and invasion of PTC cells.** A. *In vitro* scratch assay results demonstrating significantly reduced cell migration in PTC cell monolayers overexpressing TLE4 compared to control groups. B. Transwell invasion assay findings indicating a marked decrease in the invasive capacity of PTC cells with TLE4 overexpression.

We used an *in vitro* scratch assay to evaluate PTC cell migration by observing the closure of a “scratch” in a PTC cell monolayer over time. The findings showed that TLE4 overexpression significantly reduced cell migration compared to the control group (Figure 9A), highlighting TLE4's influence on cellular motility. Additionally, we conducted a Transwell invasion assay to assess the cells' invasive capacity, where cell movement through a matrix barrier simulates *in vivo* extracellular matrix conditions. The results consistently showed that TLE4 overexpression markedly diminished the cells' invasion ability (Figure 9B), indicating TLE4's inhibitory effect on PTC cell invasion. These findings collectively demonstrate that TLE4 overexpression significantly hampers the migration and invasion of PTC cells. This suggests the key role of TLE4 in controlling cellular behaviors crucial in PTC pathogenesis and progression.

### 3.9 The growth and metastasis inhibitory effect of TLE4 acts through suppression of the JAK/STAT pathway

**Figure 10:**
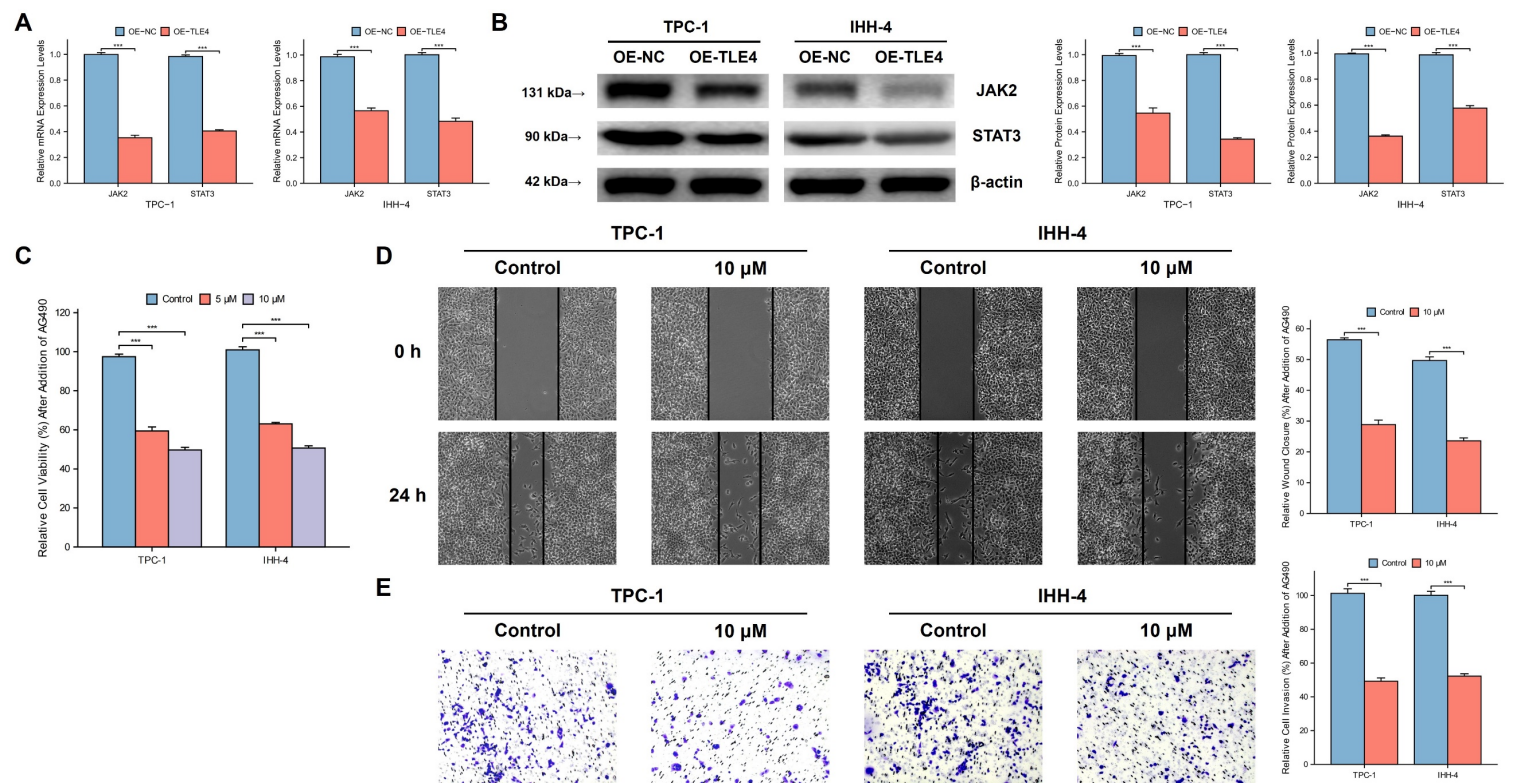
** TLE4's suppression of the JAK/STAT signaling pathway in PTC cells.** A. Quantitative PCR (qPCR) analysis showing significant downregulation of JAK2 and STAT3 mRNA levels in PTC cells overexpressing TLE4. B. Western blot analysis confirming the reduced protein expression of JAK2 and STAT3 in TLE4-overexpressed cells. C. Cell viability assays demonstrating decreased cell growth following treatment with the JAK2/STAT3 inhibitor AG490 in PTC cell lines. D. & E. The inhibitory effects of TLE4 on cell migration and invasion are paralleled by the application of AG490.

TLE4's impact on the JAK/STAT pathway, particularly highlighted in the TCGA-THCA database, was evaluated by qPCR and Western blot analysis. This ensures that the observed downregulation of JAK2 and STAT3, key mediators of this pathway, is statistically significant and not a result of random variation. The results showed significant suppression of JAK2 and STAT3 in the TLE4 overexpressed cells (Figure 10A and 10B). To validate that TLE4 overexpression inhibits PTC cell functions via the JAK/STAT pathway, we applied the JAK2/STAT3 inhibitor AG490 onto PTC cell lines and found a notable decrease in cell viability following AG490 treatment (Figure 10C), with effects extending to reduced cell migration and invasion (Figure 10D and 10E). These results suggest TLE4 primarily hinders PTC cell growth and metastasis by suppressing the JAK/STAT signaling pathway, a crucial player in cell proliferation and differentiation. The downregulation of JAK2 and STAT3, key components of this pathway, supports TLE4's role in altering PTC cell behavior.

### 3.10 Expression of TLE4 suppresses PTC tumor growth *in vivo*

**Figure 11:**
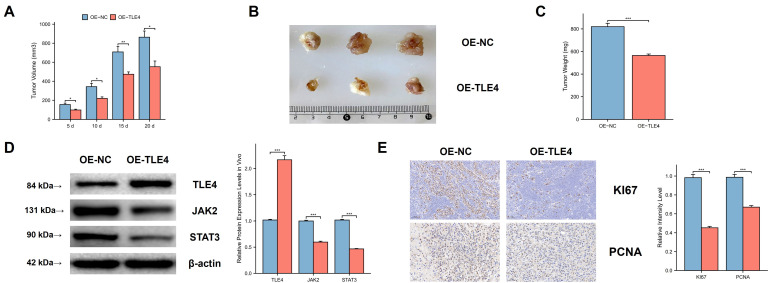
***In vivo* suppression of PTC tumor growth by TLE4 expression.** A-C. Subcutaneous xenograft models in BALB/c nude mice exhibiting significant reductions in tumor volume, size, and mass in the group overexpressing TLE4 compared to controls. D. Western blot analysis of xenograft tissues showing increased TLE4 protein levels and decreased expression of JAK2 and STAT3 in the TLE4 overexpression group. E. Immunohistochemistry results displaying reduced KI-67 and PCNA staining in tumors from the TLE4 group, indicative of lower cell proliferation rates.

We used a subcutaneous xenograft model with the PTC cell line TPC-1 in BALB/c nude mice. We observed significant differences in tumor volume, size, and mass between the TLE4 overexpressed and control groups (Figure 11A-11C), indicating TLE4's inhibitory effect on tumor growth. Western blot analysis supported this by showing increased TLE4 protein and reduced JAK2 and STAT3 proteins in the TLE4 overexpression group (Figure 11D), suggesting a mechanism for TLE4's tumor suppression. Additionally, immunohistochemistry showed decreased KI-67 and PCNA staining in the TLE4 group (Figure 11E), indicating reduced tumor cell proliferation. These results confirm TLE4's role in suppressing PTC growth *in vivo*, providing insights for its therapeutic use in papillary thyroid carcinoma.

## 4. Discussion

This research meticulously dissects the intricate landscape of gene expression variations within Papillary Thyroid Carcinoma (PTC), by harnessing the expansive repositories of data available in The Cancer Genome Atlas (TCGA) and the Gene Expression Omnibus (GEO) 32-34. This foundational effort is pivotal for delineating the genetic underpinnings of PTC, establishing a robust platform from which the molecular intricacies of the disease can be explored. The meticulous selection and analysis of 244 genes, whose expression alterations have been authenticated through a stringent validation process, spotlight pivotal elements that might be instrumental in PTC's etiology. The employment of advanced bioinformatics tools in this endeavor not only bolsters the credibility of these insights but also lays the groundwork for future investigations into the therapeutic and diagnostic implications of these genes.

The application of Weighted Gene Co-expression Network Analysis (WGCNA) in this study is particularly laudable 35, 36. To elucidate further on the mechanistic insights into how TLE4 modulates immune cell infiltration and its involvement in key signaling pathways, it's imperative to delve into the molecular dynamics facilitated by TLE4's interaction within the cellular milieu of PTC. TLE4, acting through its suppression or activation in various contexts, appears to orchestrate a delicate balance in immune surveillance within the tumor microenvironment. This balance is crucial for the maintenance or disruption of cellular homeostasis, thereby influencing tumor progression or regression. The detailed mechanism, while partially unraveled, suggests that TLE4 modulates the recruitment and differentiation of immune cells by interacting with specific cytokines and chemokines, subsequently influencing the JAK/STAT pathway. This pathway is known for its pivotal role in cell proliferation, apoptosis, and immune response regulation, highlighting the dual role of TLE4 as both a potential suppressor and promoter of tumorigenesis, depending on its regulatory context and interaction with the microenvironment.

This technique has substantially deepened our comprehension of the genetic framework of PTC, associating specific gene modules, notably the grey modules, directly with the pathology of PTC. This correlation illuminates their potential utility as innovative markers for therapeutic or prognostic purposes. By amalgamating gene expression data with clinical characteristics, the study offers a holistic view of the genetic drivers of PTC, paving the way for the development of bespoke treatment modalities. Additionally, the study's foray into precision medicine, facilitated by the deployment of machine learning algorithms, represents a significant leap forward 37-39. The pinpointing of TLE4 as a paramount biomarker through these computational techniques underscores the potency of such methods in identifying potential biomarkers in the labyrinthine nature of diseases like PTC. The specific emphasis on TLE4, corroborated by its clinical significance in the TCGA-THCA database, accentuates its utility in the diagnostic and prognostic realms of thyroid carcinoma. This aberrant activation is evidenced by increased phosphorylation of STAT3, a key mediator in the JAK/STAT pathway, which subsequently translocates to the nucleus to promote the expression of genes associated with cell proliferation and survival.

The research further delineates a crucial association between TLE4 expression and immune cell infiltration in PTC, a revelation that opens up new vistas on the influence of the tumor microenvironment in the disease's progression and its therapeutic conquests 40-43. The nuanced insights afforded by sophisticated bioinformatics analyses reveal TLE4's bifurcated impact on the genetic and immunological facets of PTC, an understanding imperative for the crafting of all-encompassing therapeutic strategies. Moreover, the detailed GO and KEGG pathway analyses uncover pivotal biological pathways and processes linked to TLE4 expression in PTC. The conspicuous connection with pathways such as the JAK-STAT signaling and cytokine-cytokine receptor interaction not only highlights TLE4's vital role in PTC's cellular dynamics but also its potential involvement in neuroendocrine activities within thyroid carcinoma, as hinted by the neuroactive ligand-receptor interaction pathway, suggesting novel research avenues in the study of PTC 44-46.

Empirical corroboration through quantitative PCR (qPCR) and Western blot analyses affirms TLE4's downregulation in PTC, signifying its conceivable role as a tumor suppressor and establishing a basis for subsequent investigations into its functional ramifications. The reproducibility of these findings has been a cornerstone of our methodology, underscored by the execution of independent validation cohorts and the application of stringent statistical analyses, ensuring the robustness and reliability of our conclusions. This empirical verification serves as a crucial bridge, linking theoretical conjectures to tangible therapeutic applications in cancer treatment. The influence of TLE4 on PTC cell proliferation, migration, and invasion, alongside its regulatory effect on the JAK/STAT pathway, underscores its significance in PTC pathogenesis and its viability as a therapeutic target. The validation of TLE4's tumor-suppressive effects *in vivo* further solidifies its role in PTC therapeutic strategies.

Considering the pivotal role of TLE4 in regulating the JAK/STAT pathway and its consequential impact on PTC progression, strategic modulation of TLE4 expression presents a novel therapeutic avenue. Therapeutic strategies aimed at restoring TLE4 expression or mimicking its function could potentially inhibit the JAK/STAT pathway, thereby suppressing the malignant characteristics of PTC cells. Such strategies could involve the use of gene therapy to increase TLE4 expression or the development of small molecules that enhance TLE4's interaction with its target DNA sequences, thereby reinstating its repressive function on JAK/STAT pathway activation. Additionally, considering the role of TLE4 in immune evasion, therapies that augment TLE4 expression may also enhance the immunogenicity of PTC cells, making them more susceptible to immune-mediated destruction. This dual mechanism of action makes TLE4 an attractive target for comprehensive therapeutic strategies that not only halt tumor growth but also engage the immune system in the eradication of cancer cells. In light of these considerations, future research should focus on identifying and characterizing agents capable of modulating TLE4 expression or function, thereby providing a foundation for the development of targeted therapies for PTC.

In considering the therapeutic viability and specificity of targeting TLE4, it is crucial to approach these results with a measure of caution. The complex nature of PTC, characterized by diverse genetic and epigenetic alterations, necessitates a careful evaluation of potential unintended effects and resistance mechanisms that may emerge from targeted interventions. Future studies aimed at validating these findings in broader patient cohorts and elucidating the potential for adverse effects are essential. The ambition to bridge bioinformatics insights with clinical application holds great promise but requires a grounded approach that carefully weighs the therapeutic benefits against the potential for off-target effects and patient heterogeneity in response to treatment.

In conclusion, this exhaustive investigation, through its synthesis of bioinformatics, machine learning, and empirical methodologies, furnishes a comprehensive understanding of PTC, elevating TLE4 to a position of critical molecular significance within the disease. The study propels forward the methodologies for treating PTC, advocating for the adoption of molecularly targeted therapies in cancer treatment. By amalgamating diverse scientific techniques, the research not only amplifies our understanding of PTC but also sets a benchmark for future oncological studies, emphasizing the import of a multidisciplinary tack in unraveling cancer's complexities and formulating efficacious treatment regimens.

## Supplementary Material

Supplementary Table 1. Comprehensive list of differentially expressed genes in papillary thyroid carcinoma across multiple databases.
